# The role of cardiac magnetic resonance imaging in the assessment of right ventricular function in patients with pulmonary arterial hypertension

**DOI:** 10.1186/1532-429X-17-S1-P364

**Published:** 2015-02-03

**Authors:** Sophia-Anastasia Mouratoglou, Alexandros Kallifatidis, George Giannakoulas, Julia Grapsa, Vasileios Kamperidis, Georgia Pitsiou, Ioannis Stanopoulos, Stavros Hadjimiltiades, Haralambos Karvounis

**Affiliations:** 1 St.Luke's Hospital, Thessaloniki, Greece; 2 1st Cardiology Department, AHEPA University Hospital, Thessaloniki, Greece; 3 Cardiology, Hammersmith Hospital, Imperial College NHS Trust, London, Thessaloniki, Greece; 4 Respiratory Failure Unit, "G.Papanikolaou" General Hospital, Thessaloniki, Greece

## Background

Cardiac Magnetic Resonance (CMR) is the gold standard technique for the assessment of right ventricular (RV) function. There is, however, scarce data on its use in the assessment of patients with pulmonary arterial hypertension (PAH). The aim of our study is to reveal the potential value of CMR in the evaluation of RV dysfunction as expressed by the pulmonary and the tricuspid annular plane systolic excursion (PAPSE and CMR-TAPSE respectively).

## Methods

All patients underwent CMR (Avanto Siemens 1,5T) and echocardiographic study at the same day. A routine set of LV and RV short-axis cines of 7mm slice thickness were acquired from base to apex using a breath-hold retrospective vector cardiography-gated balanced steady state free precession (SSFP) gradient echo sequence. Left ventricular eccentricity index in end-systole (LVSei) and end-diastole (LVDei) were defined in the short-axis view at the level of papillary muscles. PAPSE and CMR-TAPSE were defined in the RVOT and 4-chamber view respectively, by measuring the straight-line distance travelled by the lateral pulmonary and tricuspid annulus respectively, from end-diastole to end-systole. RV ejection fraction (RVEF) was obtained according to Simpson's rule. Echo- TAPSE was assessed in M-Mode view by echocardiography.

## Results

Our study included 29 patients with idiopathic PAH (22 women, mean age 49.7±14.3 years). A direct linear correlation between PAPSE and RVEF (r= 0.463, p<0.011), LVSei (r= 0.402, p<0.005), LVDei (r= 0.426, p=0.003), and echo-TAPSE (r= 0.415, p<0.011) as well as between CMR-TAPSE and echo-TAPSE (r= 0.501, p=0.002), RVEF (r=0.491, p=0.02), LVSei (r=-0.732 p=0.002) and LVDei (r=-0.625 p=0.01) was observed.

## Conclusions

CMR is a useful tool for the non invasive and reproducible evaluation of RV function, volume and pressure overload of PAH patients.

## Funding

Hellenic Cardiological Society.

**Figure 1:**
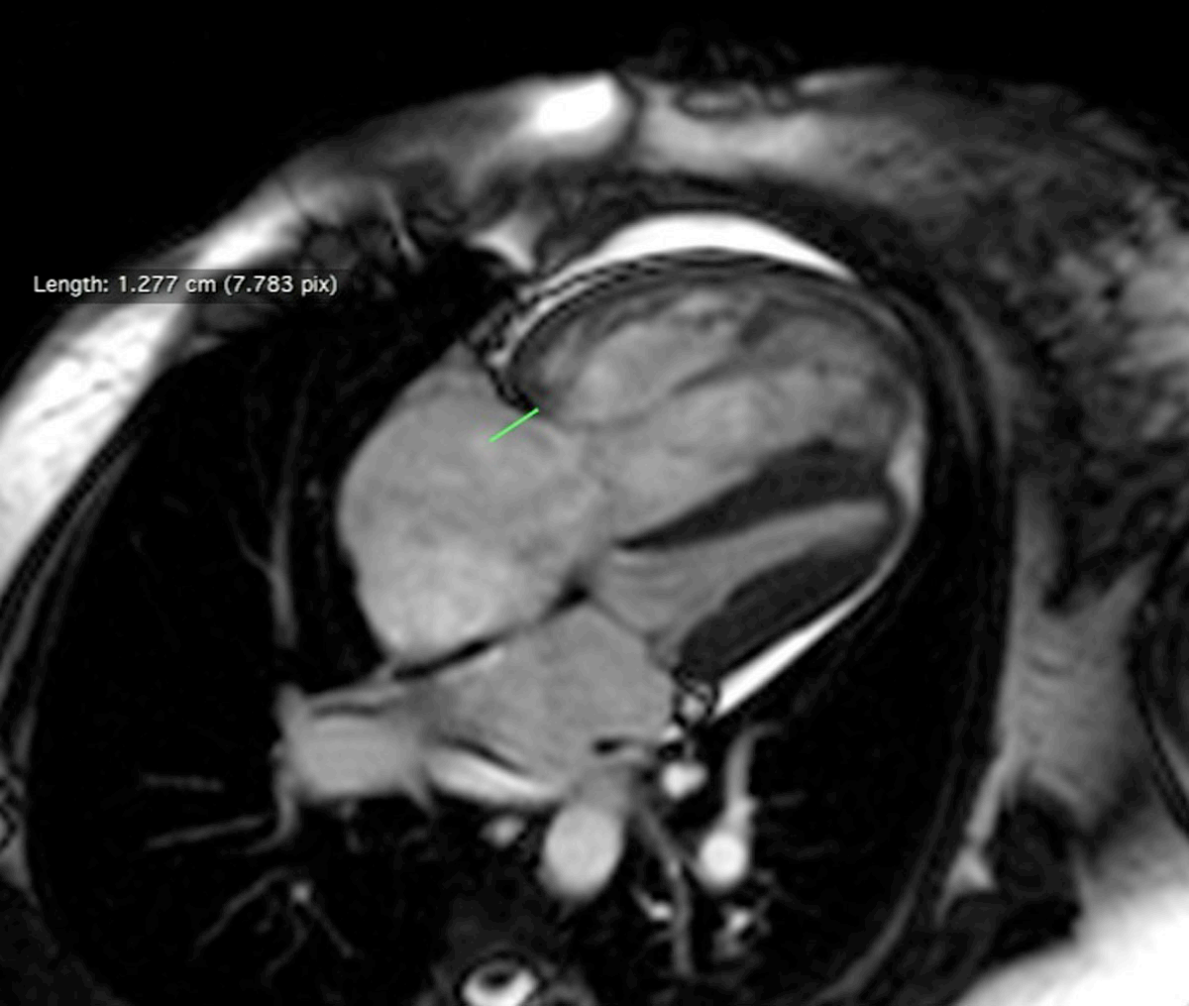


**Figure 2:**
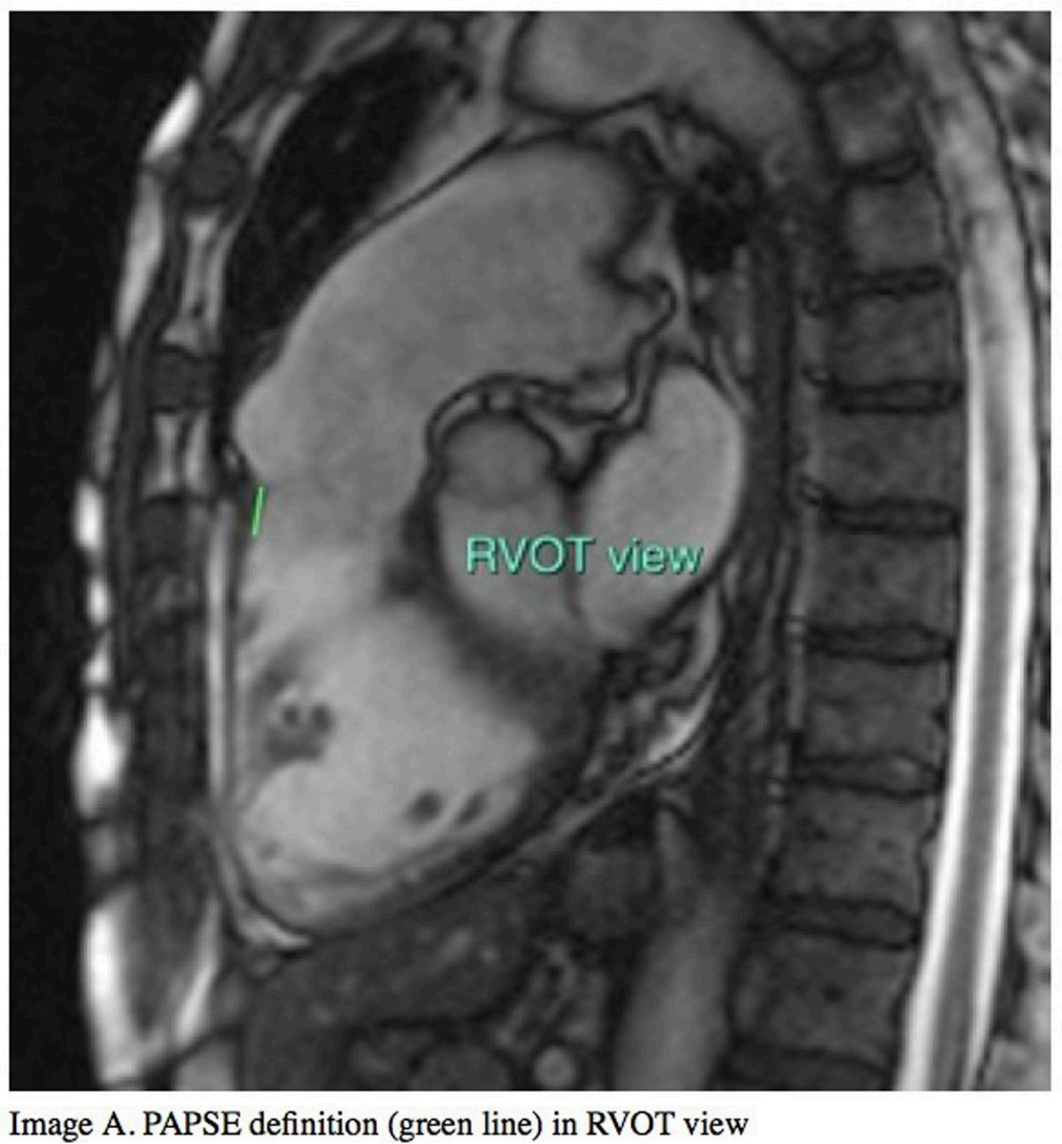
PAPSE definition (green line) in RVOT view.

